# 1796. Use of SARS-CoV-2 Antibody Tests by U.S. Infectious Disease Physicians: Qualitative Results of an Emerging Infections Network Survey, March 2022

**DOI:** 10.1093/ofid/ofad500.1625

**Published:** 2023-11-27

**Authors:** Scott S Santibanez, Adi Gundlapalli, Susan E Beekmann, Timothy M Uyeki, Panayampalli Satheshkumar, Ian D Plumb, Corinne David-Ferdon, Philip M Polgreen, L Clifford McDonald, Jefferson M Jones

**Affiliations:** CDC, Atlanta, Georgia; U.S. Centers for Disease Control and Prevention, Atlanta, Georgia; University of Iowa, IOWA CITY, Iowa; CDC, Atlanta, Georgia; CDC, Atlanta, Georgia; Division of Foodborne, Waterborne, and Environmental Diseases, Centers for Disease Control and Prevention, Atlanta, GA, Atlanta, Georgia; U.S. Centers for Disease Control and Prevention, Atlanta, Georgia; University of Iowa Carver College of Medicine, Iowa City, IA; CDC, Atlanta, Georgia; CDC, Atlanta, Georgia

## Abstract

**Background:**

Although clinical applications of SARS-CoV-2 antibody tests during the COVID-19 pandemic were limited to identifying recent/prior infection, how these tests were used for clinical management of COVID-19 patients is unknown. We consider US infectious disease (ID) physicians’ perceptions about SARS-CoV-2 antibody tests to inform preparedness for future events.

**Methods:**

In March 2022, we surveyed members of the Emerging Infections Network (EIN), a national network of ID physicians on use of SARS-CoV-2 antibody assays, interpretation of results, and clinical scenarios for which such tests were considered. We analyzed comments provided in a free text field for key themes.

**Results:**

Overall, 96 respondents provided non-mutually exclusive free-text comments. Fourteen used serology to assess patients with suspected MIS-C. Another 8 used serology in individuals with immunocompromising conditions, including organ transplant recipients and those with human immunodeficiency virus (HIV) infection, for example, when making decisions to utilize anti-SARS-CoV-2 mAbs (available at the time of the survey but no longer authorized for PReP or treatment.) Twenty-one respondents shared that the most important need for SARS-CoV-2 antibody assays was to discern a correlate of immune protection. Twenty-six considered that serology was not useful for clinical decision-making, and 23 recognized the limitations of the antibody tests, called for more studies, and indicated that additional guidance would be beneficial, noting for example, “Many clinicians order antibody tests but have no idea how to interpret or misinterpret, so guidelines on usage and when not to use would be extremely helpful.”

**Conclusion:**

This analysis provides historical insights into challenges practicing ID physicians faced in the midst of a pandemic. During March 2022, some respondents reported use of SARS-CoV-2 antibody assays including when making treatment decisions. As diagnostic and treatment modalities continue to evolve, federal agencies, medical societies, and academic partners can consider providing guidance on appropriate use of novel tests in clinical practice for near term and future responses.

**Disclosures:**

**Philip M. Polgreen, MD**, Eli Lilly: Case adjudication for a clinical trial

